# Comparative study of four *Mystus* species (Bagridae, Siluriformes) from Thailand: insights into their karyotypic diversity

**DOI:** 10.3897/CompCytogen.v15i2.60649

**Published:** 2021-04-26

**Authors:** Pun Yeesin, Phichaya Buasriyot, Sukhonthip Ditcharoen, Patcharaporn Chaiyasan, Chatmongkon Suwannapoom, Sippakorn Juntaree, Sitthisak Jantarat, Sucheela Talumphai, Marcelo de Bello Cioffi, Thomas Liehr, Alongklod Tanomtong, Weerayuth Supiwong

**Affiliations:** 1 Department of Technology and Industries, Faculty of Science and Technology, Prince of Songkla University, Pattani Campus, Muang, Pattani 94000, Thailand; 2 Department of Biology, Faculty of Science, Khon Kaen University, Muang, Khon Kaen 40002, Thailand; 3 Department of Fishery, School of Agriculture and Natural Resources, University of Phayao, Muang, Phayao 56000, Thailand; 4 Applied Science Program, Faculty of Interdisciplinary Studies, Nong Khai Campus, Khon Kaen University, Muang, Nong Khai 43000, Thailand; 5 Department of Science, Faculty of Science and Technology, Prince of Songkla University, Pattani Campus, Mueng, Pattani 94000, Thailand; 6 Major Biology, Department of Science and Technology, Faculty of Liberal Arts and Science, Roi Et Rajabhat University, Roi Et 45120, Thailand; 7 Departamento de Genética e Evolução, Universidade Federal de São Carlos (UFSCar), Rodovia Washington Luiz Km. 235, C.P. 676, São Carlos, SP 13565-905, Brazil; 8 Jena University Hospital, Friedrich Schiller University, Institute of Human Genetics, Am Klinikum 1, D-07747, Jena, Germany

**Keywords:** Chromosomes, fluorescence in situ hybridization (FISH), karyotype, *
Mystus
*

## Abstract

Karyotypes of four catfishes of the genus *Mystus* Scopoli, 1777 (family Bagridae), *M. atrifasciatus* Fowler, 1937, *M. mysticetus* Roberts, 1992, *M. singaringan* (Bleeker, 1846) and *M. wolffii* (Bleeker, 1851), were analysed by conventional and Ag-NOR banding as well as fluorescence in situ hybridization (FISH) techniques. Microsatellite d(GC)_15_, d(CAA)_10_, d(CAT)_10_ and d(GAA)_10_ repeat probes were applied in FISH. The obtained data revealed that the four studied species have different chromosome complements. The diploid chromosome numbers (2n) and the fundamental numbers (NF) range between 52 and 102, 54 and 104, 56 and 98, or 58 and 108 in *M. mysticetus*, *M. atrifasciatus*, *M. singaringan* or *M. wolffii*, respectively. Karyotype formulae of *M. mysticetus*, *M. atrifasciatus*, *M. singaringan* and *M. wolffii* are 24m+26sm+4a, 26m+24sm+2a, 24m+18sm+14a and 30m+22sm+6a, respectively. A single pair of NORs was identified adjacent to the telomeres of the short arm of chromosome pairs 3 (metacentric) in *M. atrifasciatus*, 20 (submetacentric) in *M. mysticetus*, 15 (submetacentric) in *M. singaringan*, and 5 (metacentric) in *M. wolffii*. The d(GC)_15_, d(CAA)_10_, d(CAT)_10_ and d(GAA)_10_ repeats were abundantly distributed in species-specific patterns. Overall, we present a comparison of cytogenetic and molecular cytogenetic patterns of four species from genus *Mystus* providing insights into their karyotype diversity in the genus.

## Introduction

Bagridae are the largest family of Thai catfishes, with six genera (*Bagrichthys* Bleeker, 1857, *Batasio* Blyth, 1860, *Hemibagrus* Bleeker, 1862, *Mystus* Scopoli, 1777, *Pseudomystus* Jayaram, 1968, and *Sperata* Holly, 1939) and 28 species in Thailand. They play an important role in the national economic value of the country, as they are kept in aquaria and contribute heavily to the aquaculture industry. Most species of the genus *Mystus* are booming in aquaculture, with some of them being kept in aquaria (Vidthayanon 2005). However, several species in this family are rather morphologically similar especially during the juvenile stage that may pose difficulties for their identification. *Mystus* is a poorly diagnosed group, and they are morphologically similar and diagnostic characteristics are usually subtle (Ng 2003; Ferdous 2013).

Cytogenetic studies on Thai bagrids are quite scarce; as yet only conventional cytogenetics have been applied to determine chromosome numbers and karyotype complements. Therefore, their chromosomal evolution is not clear, even though from family Bagridae up to 45 species have been karyotyped so far. The diploid chromosome number (2n) varies between 2*n* = 44 [*Coreobagrus brevicorpus* Mori, 1936)] and 2n = 80 [*Batasio fluviatilis* (Day, 1888)]. The fundamental number (number of chromosome arms, NF) varies between 64 [for *M. tengara* (Hamilton, 1822) and *M. vittatus* (Bloch, 1794)] and 116 [for *Horabagrus brachysoma* (Günther, 1864) and *H. nigricollaris* Pethiyagoda et Kottelat, 1994] (Arai 2011).

Focusing on the genus *Mystus*, chromosomal diversity and chromosomal variations among populations can be found. The so far reported 2n for diploid chromosome numbers varies between 50 and 58 chromosomes and for NF from 64 to 110 (Table 1). Intra-specific variations of 2n were reported in *M. mysticetus* Roberts, 1992 (2n = 50, 52) (Donsakul 2002; Supiwong et al. 2014a, b) and *M. vittatus* (Bloch, 1794) (2n = 50, 54, 58) (Das and Srivastava 1973; Manna and Prasad 1974; Tripathi and Das 1980; Rishi 1981; Sharma and Tripathi 1986; Khuda-Bukhsh and Barat 1987; John et al. 1992; Choudhury et al. 1993; Ramasamy et al. 2010). The cytogenetic characterization of a species could be applied to other fields such as systematics, but also economic interests, as breeding practices of organisms by using chromosome set management (Na-Nakhon et al. 1980), strain improvement (Sofy et al. 2008) and brood stock selection (Mengampan et al. 2004).

**Table 1.** Comparative cytogenetics of *Mystus* genus (2n = diploid chromosome number, m = metacentric, sm = submetacentric, st = subtelocentric, a = acrocentric, t = telocentric, NOR = nucleolar organizer regions, NF = fundamental number, and – = not available).

Species	2n		NF	Karyotype	NOR	Locality	Reference
*Mystus albolineatus* Roberts, 1994	56		108	28m+6sm+12st+10a	-	Thailand (Ayutthaya)	Donsakul (2000)
*M. atrifasciatus* Fowler, 1937	54		92	30m+8sm+16a	-	Thailand (Nakhon Phanom)	Magtoon and Donsakul (2009)
	54		96	24m+18sm+12st/a		Thailand (Bueng Kan)	Supiwong et al. (2014 a, b)
	**54**		**104**	**24m+26sm +4a/t**	**2**	**Thailand (Maha Sarakham)**	**Present study**
*M. bleekeri* (Day, 1877)	56		90	20m+14sm+10st+12a	-	India (Jammu)	Sharma and Tripathi (1986)
	56		102	32m+14sm+10a	-	India	Chanda (1989)
*M. bocourti* (Bleeker, 1864)	56		104	24m+18sm+6st+8a	-	Thailand (Nong Khai)	Donsakul (2000)
	56		100	22m+22sm+12st/a	-	Thailand (Sing Buri)	Supiwong et al. (2013a, 2014a, b)
*M. cavasius* (Hamilton, 1822)	58		102	18m+16sm+10st+14a	2	India (Jammu)	Sharma and Tripathi (1986); Rishi et al. (1994)
	58		108	18m+22sm+8t	-	India (Orissa)	Tripathi and Das (1980)
	58		102	14m+26sm+4st+14a	-	India (Bihar)	Khuda-Bukhsh et al. (1980)
*M. gulio* (Hamilton, 1822)	58		102	30m+12sm+2st+14a	2	India (West Bengal)	Das and Khuda-Bukhsh (2007b)
Female	58		108	12m+34sm+4st+8t	-	India (Orissa)	Choudhury et al. (1993)
Male	58		110	13m+33sm+4st+8t	-	India (Orissa)	Choudhury et al. (1993)
*M. multiradiatus* Roberts, 1992	54		98	30m+10sm+4st+10a	-	Thailand (Kanchana-buri)	Magtoon and Donsakul (2009)
	54		96	18m+24sm+12st/a	-	Thailand (Maha Sarakham)	Supiwong et al. (2014 a, b)
*M. mysticetus* Roberts, 1992	50		92	28m+14sm+8a	-	Thailand (Ayutthaya)	Donsakul (2002)
	52		100	26m+22sm+4st/a	-	Thailand (Maha Sarakham)	Supiwong et al. (2014a, b)
	**52**		**102**	**26m+24sm+2a**	**2**	**Thailand (Bueng Kan)**	**Present study**
*M. ngasep* Darshan et al., 2011	56		90	12m+22sm+8st+14t	-	India (Manipur)	Sing et al. (2013)
*M. singaringan* (Bleeker, 1846)	56		94	24m+14sm+10st+8a	-	Thailand (Nakhonsawan)	Donsakul (2001)
	**56**		**98**	**24m+18sm+14a**	**2**	**Thailand (Sing Buri)**	**Present study**
*M. tengara* (Hamilton, 1822)	54		64	10m+44a	-	India (Haryana)	Nayyar (1966)
Female	54		101	9m+38sm+7a	-	India	Rishi (1973)
Male	54	102		10m+38sm+6a	-	(Haryana)	Rishi (1973)
Female	54	97		25m+18sm +11a	-	India	Rishi and Rishi (1981)
Male	54	98		26m+18sm +10a	-	(Haryana)	Rishi and Rishi (1981)
*M. vittatus* (Bloch, 1794)	54	108		22m+26sm+6st	-	India (Orissa)	Tripathi and Das (1980)
	54	108		22m+20sm+12st	-	India (Jammu)	Sharma and Tripathi (1986)
	58	110		10m+30sm+12st+6t	-	India (Orissa)	Choudhury et al. (1993)
	54	108		20m+24sm+10st	-	India	Rishi (1981)
	50	64		14m+36a	-	India	Das and Srivastava (1973)
	58	104		16m+10sm+20st+12a	-	India (West Bengal)	Manna and Prasad (1974)
	54	106		28m+22sm+2st+2a	2	India (Orissa)	Khuda-Bukhsh and Barat (1987); John et al. (1992)
	54	78		6m+18sm+30a	-	India (Tamilnadu)	Ramasamy et al. (2010)
*M. wolffii* (Bleeker, 1851)	58	100		26m+10sm+6st+16a	-	Thailand (Tak)	Donsakul (2000)
	**58**	**108**		**30m+22sm+6a**	**2**	**Thailand (Nakhon Sri Thammarat)**	**Present study**

Conventional cytogenetics may be sufficient to identify intra- and interspecific variations and is an inexpensive approach. However, it has restrictions, and accordingly the use of molecular cytogenetic analyses plays an increasing role for more precise characterization of the structure of genomes, including that of fishes. Especially, fluorescence in situ hybridization (FISH) for mapping of repetitive DNA sequences provided important contributions to the characterization of biodiversity and evolution in divergent fish groups (Cioffi and Bertollo 2012), especially as some microsatellite repeats are species-specific (Cioffi et al. 2015). To date, there are only three studies within Bagridae using such FISH techniques, all performed by our group (Supiwong et al. 2013a, 2014a, b).

**Figure 1.**
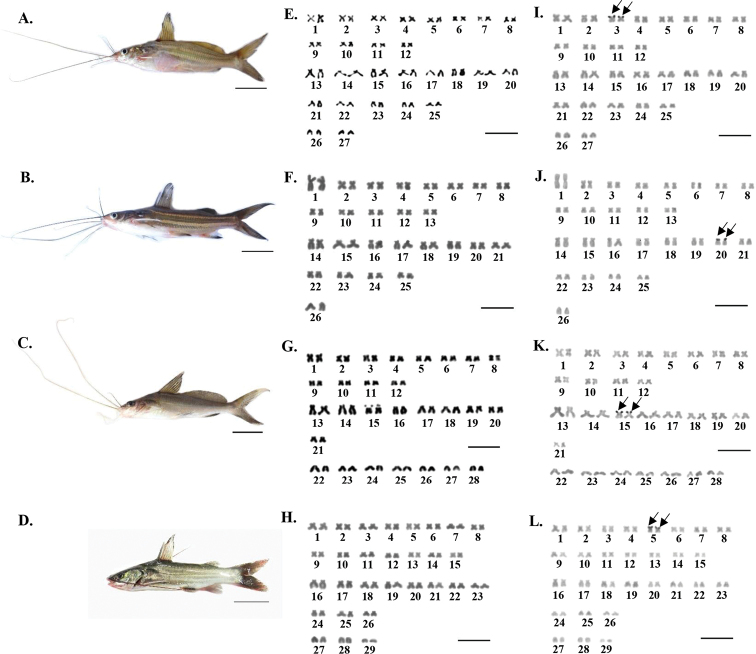
Specimens and karyotypes from conventional staining and Ag-NOR banding techniques of *Mystus atrifasciatus* (**A, E, I**), *M. mysticetus* (**B, F, J**), *M. singaringan* (**C, G, K**) and *M. wolffii* (**D, H, L**); arrows indicate NOR carrying chromosomes. Scale bars: 2 cm (**A–D**); 5 µm (**E–L**).

In the present study, chromosomal structures and genetic markers for Thai populations of *M. atrifasciatus* Fowler, 1937, *M. mysticetus*, *M. singaringan* (Bleeker, 1846) and *M. wolffii* (Bleeker, 1851) (Fig. 1A–D) were for the first time analysed by cytogenetics and molecular cytogenetics.

## Material and methods

Ten males and ten females of each species were collected from the Chi (Maha Sarakham Province), Songkhram (Bueng Kan Province), Chao Phraya (Sing Buri Province) and Pak Phanang Basins (Nakhon Sri Thammarat Province), Thailand from 2016–2018. The procedures followed ethical protocols as approved by the Institutional Animal Care and Use Committee of Khon Kaen University, based on the Ethics of Animal Experimentation of the National Research Council of Thailand ACUC-KKU-15/2559. Preparation of fish chromosomes from kidney cells was done as previously reported (Supiwong et al. 2012; Pinthong et al. 2015). The chromosomes were stained with Giemsa solution for 10 minutes. Ag-NOR banding was performed by applying two drops of 2% gelatin to the chromosomes, followed by four drops of 50% silver nitrate (Howell and Black 1980). Metaphases were evaluated according to the chromosome classification of Levan et al. (1964). Chromosomes were classified as metacentric (m), submetacentric (sm), subtelocentric (st) or acrocentric (a). Fundamental number, NF (number of chromosome arm) was obtained by assigning a value of two to metacentric and submetacentric chromosomes and one to subtelocentric and acrocentric chromosomes. The chromosome sizes were calculated applying the method of Tanomtong (2011).

Microsatellites d(GC)_15_, d(CAA)_10_, d(CAT)_10_ and d(GAA)_10_ repeat probes (Kubat et al. 2008) were directly labeled by Cy3 at 5´ ends during synthesis (Sigma, St. Louis, MO, USA). FISH under high stringency conditions on mitotic chromosome spreads (Pinkel et al. 1986) was performed as previously reported (Supiwong et al. 2017b; Yano et al. 2017). The evaluation was done on an epifluorescence microscope Olympus BX50 (Olympus Corporation, Ishikawa, Japan).

## Results

### Diploid number, fundamental number and karyotype of *Mystus atrifasciatus*, *M. mysticetus*, *M. singaringan* and *M. wolffii*

The four studied *Mystus* species have different diploid chromosome numbers (2n) and fundamental numbers (NF) as follows: the 2n (NF) were 52 (102), 54 (104), 56 (98) and 58 (108) in *M. mysticetus*, *M. atrifasciatus*, *M. singaringan* and *M. wolffii*, respectively. The karyotypes of *M. atrifasciatus* (24m+26sm+4a), *M. mysticetus* (26m+24sm+2a), *M. singaringan* (24m+18sm+14a) and *M. wolffii* (30m+22sm+6a) were species-specific (Fig. 1E–H; Table 1). Differentiated sex chromosomes between male and female specimens could not be identified in all analyzed species.

### Chromosome markers in *Mystus atrifasciatus*, *M. mysticetus*, *M. singaringan* and *M. wolffii*

**Figure 2.**
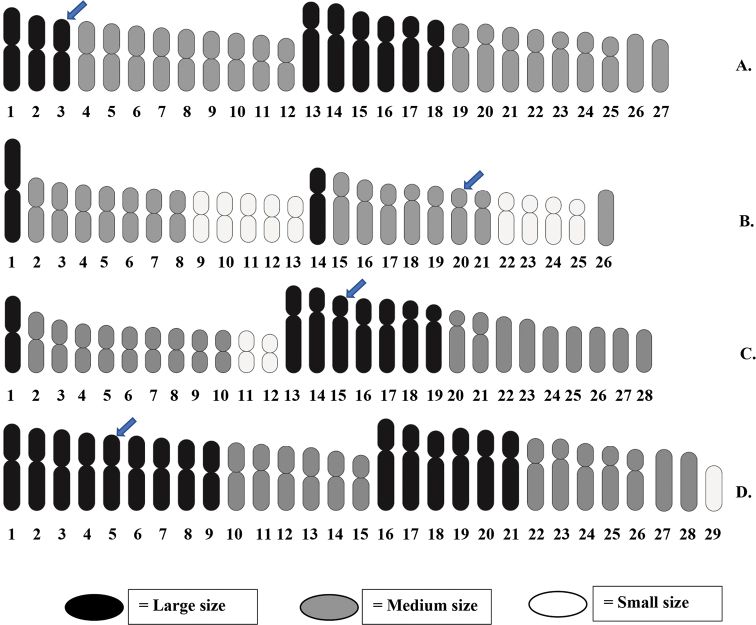
Idiograms representing shapes and sizes of chromosomes (haploid set) of **A***Mystus atrifasciatus***B***M. mysticetus***C***M. singaringan* and **D***M. wolffii*; arrows indicate NOR carrying chromosomes.

One single pair with NOR-bearing chromosomes was present in all four species analyzed. NOR positions were observed at regions adjacent to the telomere of the short arm of the chromosome pairs 3 (metacentric), 20 (submetacentric), 15 (submetacentric), and 5 (metacentric) in *M. atrifasciatus*, *M. mysticetus*, *M. singaringan* and *M. wolffii*, respectively (Figs 1I–L, 2). The typical diversity of chromosome shapes and sizes among the four analyzed species is shown in Fig. 2 and Table 2. Karyotypic complements comprise most bi-armed and few mono-armed chromosomes revealed in *M. atrifasciatus*, *M. mysticetus* and *M. wolffii* whereas in *M. singaringan*, there are several pairs of both bi-armed and mono-armed chromosomes. Chromosome sizes are classified as large (L), medium (M) and small (S) in each species as follows: 18L+36M in *M. atrifasciatus*, 4L+30M+18S in *M. mysticetus*, 16L+36M+4S in *M. singaringan*, and 30L+26M+2S in *M. wolffii*.

**Table 2.** Cytogenetic and FISH studies on four *Mystus* fishes in Thailand (2n = diploid chromosome number, NF = fundamental number or number of chromosome arm, m = metacentric, sm = submetacentric, a = acrocentric, NOR = nucleolar organizer region, I = interstitial site, T = telomere, W = whole chromosome).

*Mystus* Species	2n	NF	Chromosome type			Ag-NOR pair (type)	Microsatellite patterns			
			m	sm	a		(GC)_15_	(CAA)_10_	(CAT)_10_	(GAA)_10_
*M. atrifasciatus* Fowler, 1937	54	104	24	26	4	3 (m)	T&I	T&I	T&I	I
*M. mysticetus* Roberts,1992	52	102	26	24	2	20 (sm)	T&I	T&I	T&I	T&I
*M. singaringan* (Bleeker, 1846)	56	98	24	18	14	15 (sm)	T&I	T&I	T	T&I
*M. wolffii* (Bleeker, 1851)	58	108	30	22	6	5 (m)	W&T	T	W&T	T&I

### Patterns of microsatellite repeats in the genomes of *Mystus atrifasciatus*, *M. mysticetus*, *M. singaringan* and *M. wolffii*

**Figure 3.**
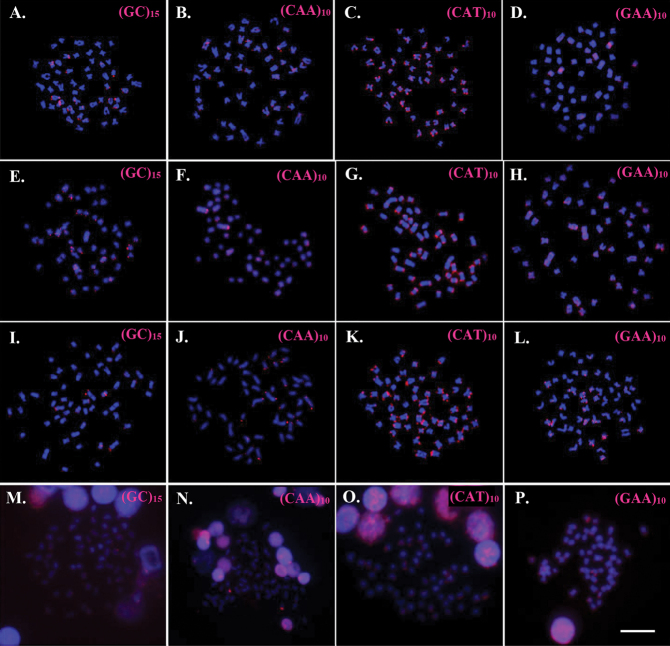
Metaphase chromosome plates showing d(GC)_15_, d(CAA)_10_, d(CAT)_10_ and d(GAA)_10_ microsatellites mapping on chromosomes of *Mystus atrifasciatus***(A, E, I, M)**, *M. mysticetus***(B, F, J, N)**, *M. singaringan***(C, G, K, O)** and *M. wolffii* (**D, H, L, P)**. Scale bars: 5 µm.

The mapping of d(GC)_15_, d(CAA)_10_, d(CAT)_10_ and d(GAA)_10_ microsatellites showed different hybridization signals among the species. The repeats of d(GC)_15_ and d(CAA)_10_ are abundantly distributed in the telomeric regions of several pairs and in interstitial sites of some chromosomes in *M. atrifasciatus*, *M. mysticetus*, *M. singaringan*. In contrast, in *M. wolffii*, d(GC)_15_ repeats are dispersed throughout all chromosomes, while d(CAA)_10_ repeats are accumulated at telomeric positions of some chromosome pairs with more density in only one pair. The d(CAT)_10_ repeats in *M. atrifasciatus* and *M. mysticetus* display high accumulations at the telomeric regions of almost all chromosomes and interstitial sites in some pairs whereas they have high accumulations at only the telomeric regions of almost all chromosomes in *M. singaringan*, and highly distributed in some chromosome pairs in *M. wolffii*. The d(GAA)_10_ repeats are abundantly distributed at interstitial and telomeric regions of several chromosome pairs in *M. mysticetus*, *M. singaringan* and *M. wolffii*, while they are highly accumulated in some chromosome pairs of *M. atrifasciatus* (Fig. 3; Table 2).

## Discussion

### Diploid chromosome numbers, fundamental numbers and karyotypes of *M. atrifasciatus*, *M. mysticetus*, *M. singaringan* and *M. wolffii*

**Figure 4.**
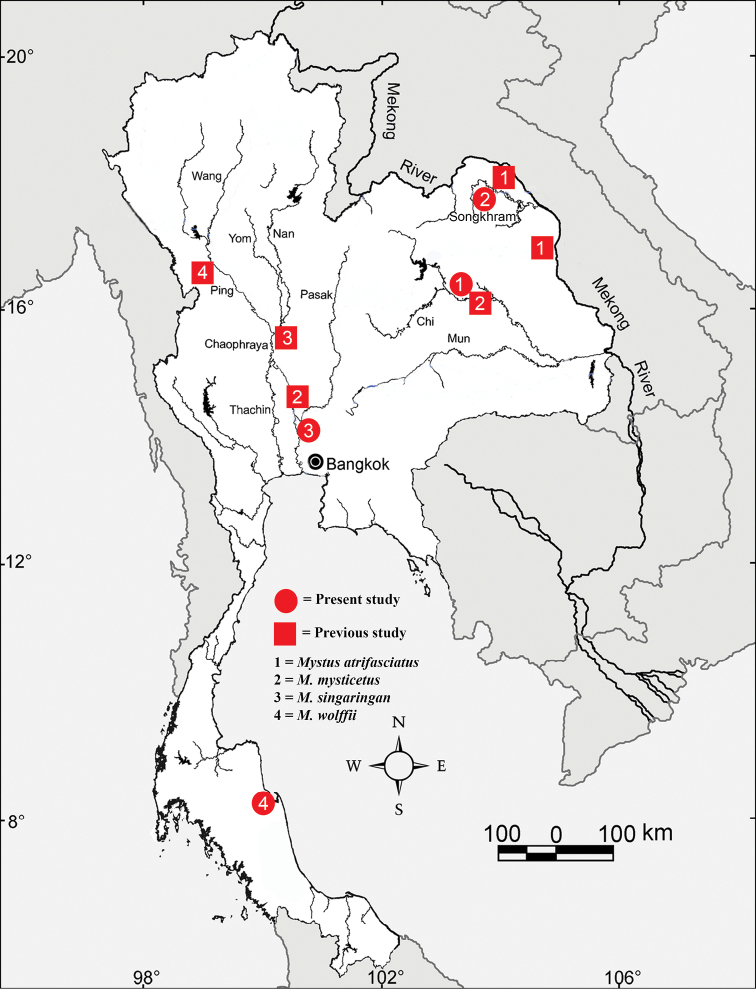
Map showing the comparison of sampling sites among present and previous studies.

The diploid chromosome numbers (2n) in all analyzed species confirmed previous cytogenetic studies (Donsakul 2000, 2001; Magtoon and Donsakul 2009; Supiwong et al. 2014a, b), except for *M. mysticetus* with 2n=50 reported in a previous study (Donsakul 2002) and 52 in the present one. In agreement with the literature, 2n in the genus *Mystus* ranges between 50 and 58 chromosomes (Arai 2011; Table 1). The possible mechanisms that promoted intra- and interspecific karyotype diversification are biogeographic barriers, small population, limited gene flow (Galetti Jr. et al. 2000). Although all studied species except *M. mysticetus*, had the same 2n as previous studies, the karyotypes were different, probably because of different sampling sites should be considered (Fig. 4). The predominant 2n in this genus is 56 chromosomes (five from 13 species) and may represent an ancestral character in this family (Sharma and Tripathi 1986). This is consistent with the hypothesis of Oliveira and Gosztonyi (2000) that 2n=56 could be a plesiomorphic character in the order Siluriformes. However, NF and karyotypes found in the present study differ from all previous reports (Donsakul 2000, 2001, 2002; Magtoon and Donsakul 2009; Supiwong et al. 2014a, b). These differences may be species-specific variations within populations, and/or misidentification of species, or different species in presumed species complexes. NF in *Mystus* vary from 64 to 110. Ghigliotti et al. (2007) suggested that species with a higher NF value are more advanced in evolutionary terms than such with lower one. That hypothesis can be described that primitive karyotype of fish possesses many acrocentric chromosomes (mono-arm chromosomes). During evolution, the mono-arm chromosomes changed to bi-arm chromosomes. The NF would be unaltered, but the 2n would decrease. Changes in NF appear to be related to the occurrence of pericentric inversions, which play a major role for karyotypic rearrangement in fishes and other vertebrates (King 1993; Galetti Jr. et al. 2000; Wang et al. 2010). Accordingly, from comparative analysis among the here studied four *Mystus* species, NF data and analyses of karyotypic complements indicate for that *M. singaringan* has the most primitive karyotype while *M. wolffii* has the most derivative karyotype. As often seen in fishes of this family, no heteromorphic sex chromosomes for males and females could be identified. Nonetheless it must be mentioned, that there are two species, *M. gulio* (Hamilton, 1822) and *M. tengara* (Hamilton, 1822), which have differentiated sex chromosome systems as XX/XY and ZZ/ZW, respectively (Arai 2011). Accordingly, differentiated sex chromosome system in this fish group seems to be a quite rare phenomenon.

Karyotypes of the genus *Mystus* in Thailand showed high diversification (Table 1). Seven species have been cytogenetically studied. The 2n ranged between 50 chromosomes in *M. mysticetus* (Donsakul 2002) and 58 chromosomes in *M. wolffii* (Donsakul 2000; present study). The predominant 2n is 56 chromosomes found in *M. albolineatus* (NF = 108, 28m+6sm+12st+10a) (Donsakul 2000), *M. bocourti* (NF = 100, 22m+22sm+12st/a; NF = 104, 24m+18sm+6st+8a) (Donsakul 2000; Supiwong et al. 2013a, 2014a, b) and *M. singaringan* (NF = 94, 24m+14sm+10st+8a; NF = 98, 24m+18sm+14a) (Donsakul 2001; present study). Our results showed differences among NFs and karyotypes in the studied species. Interestingly, *M. mysticetus* had two variants, 2n = 50 chromosomes (NF=92, 28m+14sm+8a), found in Ayutthaya Province, Central Thailand (Donsakul 2002), and 52 chromosomes (NF = 100, 26m+22sm+4st/a; NF=102, 26m+24sm+2a) found in Maha Sarakham and Bueng Kan Provinces, Northeast of Thailand (Supiwong et al. 2014a, b; present study) (Fig. 4). This variation may be caused by a rearrangement of chromosomes by centric fusion and pericentric inversion during chromosomal evolution in groups of populations separated by a geographic barrier.

### Chromosome markers for *M. atrifasciatus*, *M. mysticetus*, *M. singaringan* and *M. wolffii*

#### Nucleolus organizer regions (NORs)

The localization of nucleolus organizer regions (NORs) is a simple method to determine chromosomal marker. NORs are specific positions on the chromosome that consist of tandemly repeated sequences of ribosomal genes (rRNA). In eukaryotes, each unit is composed of three genes coding for 18S, 5.8S and 28S ribosomal RNA (Sharma et al. 2002). Generally, most fishes have one pair of small NORs (single NOR) on chromosomes. However, some species of fishes have more than two NORs which may be caused by the translocation between some part of the chromosome with NORs and another chromosome (Sharma et al. 2002). Interspecific and intraspecific NOR polymorphism in the number of NORs per genome, in the chromosomal location of NOR sites, in the relative sizes of individual NORs, and in the number of active NOR sites per cell are commonly observed in fish, where the rDNA loci have been shown to be highly dynamic (Milhomem et al. 2013). Changes in chromosome number and structure can alter the number and structure of NOR as well. The pattern of NORs may be specific to populations, species and subspecies. Robertsonian translocations may cause losses of NOR. Species, which have limited gene exchange due to geographical isolation, have elevated karyotype numbers and NOR variation. (Yüksel and Gaffaroğlu 2008). The NOR is frequently used to compare variations as well as to identify and explain specifications. Therefore, it can be used as taxonomic and systematic characters in order to infer phylogenetic hypotheses of species relationships (Gold 1984; Amemiya and Gold 1990).

If these loci are active during the interphase before mitosis, they can be detected by silver nitrate staining (Howell and Black 1980). The single NOR-bearing chromosome pairs in the present study is consistent in *M. cavasius* (Hamilton, 1822) (Sharma and Tripathi 1986; Rishi et al. 1994), *M. gulio* (Das and Khuda-Bukhsh 2007b), and *M. vittatus* (Khuda-Bukhsh and Barat 1987; John et al. 1992). This character is a common characteristic found in many species in this family such as *Bagrichthys majusculus* Ng, 2002 (Supiwong et al. 2018), *He. menoda* (Hamilton, 1822) (Barat and Khuda-Bukhsh 1986), *He. wyckii* (Bleeker, 1858) (Supiwong et al. 2017c), *Horabagrus brachysoma* (Günther, 1864) (Nagpure et al. 2003), *Ho. nigricollaris* Pethiyagoda et Kottelat, 1994 (Nagpure et al. 2004), *Pelteobagrus ussuriensis* (Dybowski, 1872) (Kim et al. 1982), *Pseudobagus vachelii* (Ueno 1985), *Pseudomystus siamensis* (Regan, 1913) (Supiwong et al. 2013b), *Rita rita* (Hamilton, 1822) (Khuda-Bukhsh and Barat 1987) and *Sperata seenghala* (Sykes, 1839) (Sharma and Tripathi 1986; Das and Khuda-Bukhsh 2007a). However, only a single species, *Tachysurus fulvidraco* (Richardson, 1846), has two NOR carrying chromosome pairs (Zhang et al. 1992). In fishes, a single NOR carrying chromosome pair is considered as a primitive state (Milhomem et al. 2013). Many families such as Chaetodontidae (Supiwong et al. 2017a), Lutjanidae (Phimphan et al. 2017), Notopteridae (Maneechot et al. 2015), Scaridae (Kaewsri et al. 2014), Serranidae (Pinthong et al. 2013), share this character. Also, for fishes the location of NORs in a terminal position, as seen in the studied species, is also considered as a primitive characteristic (Vitturi et al. 1995).

#### Patterns of microsatellite repeats on the genomes of *Mystus atrifasciatus*, *M. mysticetus*, *M. singaringan* and *M. wolffii*

Repetitive DNAs like microsatellites can be used to spot genomic evolution as previously been reported for different fish groups (Cioffi et al. 2010; Cioffi and Bertollo 2012; Terencio et al. 2013; Yano et al. 2014; Cioffi et al. 2015; Moraes et al. 2017, 2019; Sassi et al. 2019). It is known from fossil records that there is a major evolutionary diversification in Siluriformes fishes; this has in parts already also been verified at chromosomal level.

Here, four bi- and tri-nucleotide microsatellite sequences were mapped on chromosomes of four *Mystus* species. The patterns of microsatellites d(GC)_15_ and d(CAA)_10_ repeats in three species in the present study (*M. atrifasciatus*, *M. mysticetus*, *M. singaringan*) are similar to those found in *Channa micropeltes* (Cuvier, 1831) (Cioffi et al. 2015). On the other hand, they are differences known for *C. gachua* (Hamilton, 1822), *C. lucius* (Cuvier, 1831), *C. striata* (Bloch, 1793) (Cioffi et al. 2015), *Toxotes chatareus* (Hamilton, 1822) (Supiwong et al. 2017b) and Asian swamp eel, *Monopterus albus* (Zuiew, 1793) (Supiwong et al. 2019). The pattern of microsatellite d(GC)_15_ repeats in *M. wolffii* is similar to that of *C. lucius* (Cioffi et al. 2015) and *T. chatareus* (Supiwong et al. 2017a). Interestingly, the patterns of microsatellite d(CAT)_10_ repeats in *M. atrifasciatus*, *M. mysticetus* and *M. singaringan* are similar to the patterns of the (CA)_15_ repeats on chromosomes of other species in the family Bagridae (Supiwong et al. 2013a, 2014b). Comparative study on four species showed that not only there are differences of 2n, NF and karyotype, but the patterns of microsatellite repeat on chromosomes also have difference among them. Thus, the cytogenetic data may be a tool for classification of fish species that there is similar morphology as the stripe *Mystus* (*M. atrifasciatus* and *M. mysticetus*).

From previous reports, it may be carefully deduced that most heterochromatin in fish genomes consist of microsatellites (Cioffi and Bertollo 2012). However, microsatellites have also been found in non-centromeric regions, many of them were located either near or within genes (Rao et al. 2010; Getlekha et al. 2016). Indeed, GC rich motifs are common in exons of all vertebrates (Chistiakov et al. 2006). Since higher recombination rates can be found near the telomeric region (Jensen-Seaman et al. 2004), it is possible that the physical proximity of microsatellite and rDNA repeats could favor the evolutionary spreading of both sequences together, despite the possibility of spreading some errors, too. Repetitive DNA sequences could act as primary driving forces in speciation (Biémont and Vieira 2006). These sequences are closely associated with heterochromatic regions, thus contributing to gene activation and structural maintenance of chromosomes (Dernburg et al. 1996). Therefore, great variations in the amount and position of these sequences could create fertility barriers by fostering the occurrence of chromosomal rearrangements (Cioffi and Bertollo 2012).

Indeed, the distribution of microsatellite motifs in fish genomes could be biased to some specific noncoding regions, as found in the Asian swamp eel, *M. albus* (Li et al. 2017). Finally, closely related fish species involved in recent speciation events could present a differential pattern in the distribution and quantity of microsatellite sequences on chromosomes, as demonstrated for naked catfishes (Supiwong et al. 2014b), channid fishes (Cioffi et al. 2015) and four *Mystus* in the present study.

## Conclusions

The present research is the first report on NOR and microsatellites d(GC)_15_, d(CAA)_10_, d(CAT)_10_ and d(GAA)_10_ mapping in *M. atrifasciatus*, *M. mysticetus*, *M. singaringan* and *M. wolffii*. There are differences in the diploid chromosome number, the fundamental numbers, karyotypes, pairs having NORs, and patterns of microsatellite distributions on chromosomes. These results indicated that (molecular) cytogenetic data can be used for classification in related fish species and to explain karyotype diversification.
